# Multidrug-resistant Intestinal *Staphylococcus aureus* among Self-medicated Healthy Adults in Amassoma, South-South, Nigeria

**DOI:** 10.3329/jhpn.v29i5.8898

**Published:** 2011-10

**Authors:** Adebola Onanuga, Tarilate C. Temedie

**Affiliations:** Department of Pharmaceutical Microbiology and Biotechnology, Faculty of Pharmacy, Niger Delta University, Wilberforce Island, Bayelsa State, Nigeria

**Keywords:** Antimicrobial agents, Drug resistance, Microbial, Gastrointestinal tract, Self-medication, *Staphylococcus aureus*, Nigeria

## Abstract

Multiple antibiotic resistant *Staphylococcus aureus* is one of the common causes of severe nosocomial infections, and the gastrointestinal tract is an important source of its transmission. This study assessed the previous usage of antibiotics by healthy adults (university students and villagers) in Amassoma, Nigeria, and investigated the antimicrobial resistance patterns of their intestinal *S. aureus* isolates. A questionnaire was used for evaluating the previous usage of antibiotics by the volunteers. Stool samples were collected and cultured, and *S. aureus* isolates were confirmed using standard microbiological protocols. Their antimicrobial resistance patterns were determined using disc-diffusion and agar dilution techniques. In total, 54 (45.0%) volunteers used antibiotics on self-medications, and the practice was significantly higher (p=0.01) among the villagers than among the students. The level of judicious use of prescribed antibiotics was significantly higher (p=0.003) among the students than among the villagers. Thirty-eight (31.7%) healthy adults were colonized with intestinal *S. aureus.* The percentages of resistance of the isolates to some antibiotics were as follows: ampicillin–68.4%, doxycycline–60.5%, cefoxitin–34.2%, vancomycin–36.8%, erythromycin–34.2%, and gentamicin–5.3%. Twenty-five (65.8%) of the isolates were multidrug-resistant. The need for sound education on the appropriate use of antibiotics and the importance of proper personal hygiene as means of controlling the spread of bacterial antibiotic resistance are highlighted. Thus, effective strategies in these areas are strongly recommended.

## INTRODUCTION

*Staphylococcus aureus,* the golden cluster seed, is a spherical bacterium frequently found in the nose, throat, intestine, vagina, and skin of human body (1). It is a pathogen of greater concern because of its ability to cause a diverse array of life-threatening infections and its capacity to adapt fast to the different environmental conditions (2,3). These features have made infections of *S. aureus* increasingly difficult to treat because of the fast rate at which it develops resistance to common antimicrobial agents.

Multiple antibiotic resistance is a major health concern in the treatment of staphylococcal infections, especially infections of methicillin-resistant *S. aureus* (MRSA) which occurs due to the extensive use of antimicrobial agents, coupled with the transmission of an appreciable proportion of the organism by person-to-person contacts (4). Hence, effective control of antibiotic use and prevention of the transmission of these strains are essential to eradicate this infectious organism.

The gut is an important habitat of parasites and bacteria which can be transmitted through objects contaminated with faeces, indicating the importance of faecal-oral transmission in humans, which can lead to mild or severe diseases in susceptible individuals or when found in sterile sites of the body (5). The presence of staphylococci in stools has been recognized as an important pathogen responsible for antibiotic-associated diarrhoea in humans (6). Reports of recent studies are also implicating the gut as an important reservoir of antibiotic-resistant *S. aureus* strains (7,8).

Most studies on *S. aureus* have so far been conducted on samples from the nose and throat but only a very few detailed studies on its colonization of the gut have been reported. Thus, there is a need for more studies on *S. aureus* from the gut, as an important reservoir of multiple antibiotic-resistant bacterial strains, especially in developing countries where the control of antibiotic use is inadequate. We report here the prevalence of multidrug-resistant faecal *S. aureus* isolates from healthy inhabitants in Amassoma, Nigeria.

## MATERIALS AND METHODS

### Study subjects

In total, 120 subjects comprising an equal number of villagers and students of the Niger Delta University, Wilberforce Island, Amassoma, Bayelsa state, Nigeria, were randomly recruited into the study for three months from March 2009. Amassoma is a very small village in Bayelsa state, south-southern Nigeria, where the new growing state university exists. Trading, farming of crops, and fishing are the occupations of the villagers.

Willingness of the subjects to participate in the study was a strong criterion for the study. The volunteers gave informed consents by providing demographic data and a completed questionnaire on the evaluation of their previous use of antibiotics. They were not on any antibiotic for at least two weeks at the time of the sampling nor had been admitted to any hospitals in the last one year before the survey. They were aged 15-35 years.

### Sampling and isolation of ***S. aureus***

Sterile cotton swabs were given to the volunteers to deep into their freshly-produced faeces and return into the swab's case. These stool samples were collected from the volunteers within 24 hours and transported within an hour to the laboratory in iced packs, where these were inoculated directly onto prepared sterilized mannitol salt agar (MSA) plates (Oxoid, UK) and streaked before incubated at 37 °C for 24 hours.

### Identification of ***S. aureus***

*S. aureus* was identified and differentiated from related organisms based on the colony morphology on the MSA plates. Two discrete yellow colonies on each of the MSA plates were subcultured on sterile nutrient agar (Oxoid, UK) slants aseptically and incubated at 37 °C for 24 hours. The proliferated organisms were characterized by Gram staining, productions of catalase, DNase, and coagulase (using human plasma). The organisms that were Gram-positive clustered-shaped cocci, positive to catalase, DNase and coagualse tests were confirmed as *S. aureus*. These were stored on slant nutrient agar at 4 °C and were used for antibiotic-susceptibility testing.

### Antibiotic-susceptibility testing

#### Agar diffusion method (zone of inhibition measurement)

Each isolate of *S. aureus* was standardized using colony suspension method, and the strain's suspension was matched with 0.5 McFarland standards to give a resultant concentration of 1.5×10^8^ cfu/mL (colony-forming unit per millilitre) (9). The susceptibility testing of commonly-used antibiotics was determined using the modified Kirby-Bauer diffusion technique by swabbing each of the Mueller–Hinton agar (Oxoids U.K) plates with the resultant saline suspension of each strain, and the following six antibiotic discs from Oxoids, UK, were placed on the plate after 20 minutes of inoculation: cefoxitin 30 μg (as a substitute for methicillin) (9), gentamicin 10 μg, ofloxacin 5 μg, ciprofloxacin 5 μg, doxycycline 30 μg, and trimethoprim/sulphamethoxazole (co-trimoxazole) 1.25/23.75 μg (10). The plates were then allowed to stand for at least 30 minutes before incubated at 30 °C for 24 hours to favour the growth of any methicillin-resistant strains (11). The diameter of the zone of inhibition produced by each antibiotic disc after incubation was measured and interpreted using the zone diameter interpretative standards of the Clinical and Laboratory Standard Institute (CLSI) (9).

#### Agar dilution method (MIC test)

Standardized solutions of six other commonly-used antibiotics—vancomycin (Novaplus, USA), ampicillin (Merck, Germany), erythromycin (Merck, Germany), chloramphenicol (Merck, Germany), amoxicillin-clavulanic acid (augmention) (Glaxowellcome, UK), and cefuroxime (Glaxowellcome, UK)—were aseptically prepared. These solutions were used for preparing Mueller-Hinton agar plates of breakpoints of varying CLSI minimum inhibitory concentrations (MICs): vancomycin 2-12 μg/mL, cefuroxime 8 μg/mL, ampicillin 0.25 μg/mL, erythromycin 0.5 μg/mL, chloramphenicol 8 μg/mL, and amoxicillin-clavulanic acid (augmentin) 4/2 μg/mL. The plates were spot-inoculated with each strain's standardized suspension and incubated at 30 °C for 24 hours (11). Strains that showed growth on each of the antibiotic agar plates were regarded as resistant to the antibiotic using the CLSI breakpoint standards (9).

### Statistical analysis

Frequencies were obtained, and percentages were calculated for the study variables. The demographic characteristics were compared using the chi-square test (two-tailed) and using the SPSS software (version 15). All reported p values were two-sided, and p value of less than or equal to 0.05 (p≤0.05) was considered significant.

## RESULTS

### Analysis of questionnaire

Copies of the questionnaire were retrieved from all the study subjects. Twenty-nine (24.2%) of them were aged 15-19 years, 65 (54.1%) were aged 20-25 years, and 26 (21.7%) were aged above 25 years. Fifty-four (45%) of the subjects (students and villagers) used antibiotics on self-medications, of whom 34 (63%) were villagers while the remaining 20 (37%) were students (Table 1). The observed difference in the use of antibiotics between the two groups was significant (p=0.01). Twenty-six (43.3%) students and 32 (53.3%) villagers used antibiotics for reasons other than treatment of diseases (Table 2), the difference of which was not significant (p=0.273). Twenty-five (41.7%) students and 10 (16.7%) villagers judiciously used their prescribed antibiotics (Table 3), the difference of which was highly significant (p=0.003). The distribution of the antimicrobial agents used on self-medication by the subjects is shown in the Figure.

**Table 2.** Reasons for antibiotic use by study subjects

Reason	Students (n=60)		Villagers (n=60)	
	No.	%	No.	%
Without any reason	0	0.0	4	6.7
Prevention of infection only	10	16.7	17	28.3
Treatment of diseases only	34	56.7	28	46.7
Both prevention and treatment	16	26.7	11	18.3

The p value for reasons other than treatment of diseases only in both the groups=0.273

### Prevalence of ***S. aureus***

In total, 38 (31.7%) isolates of *S. aureus* were detected from 120 stool samples screened, with 18 (30.0%) from the villagers and 20 (33.3%) from the students (Table 4). The observed differences in the yield of the organism between the two groups of subjects and between their sexes were not significant (p>0.05).

### Antimicrobial resistance profile

The isolates generally showed high resistance to ampicillin, doxycycline, co-trimoxazole, erythromycin, and chloramphenicol. However, low levels of resistance were exhibited by the isolates to cefuroxime, augmentin, ciprofloxacin, ofloxacin, and gentamicin. The differences observed in bacterial resistance to various antimicrobial agents between the two groups of subjects were not significant (p>0.05) (Table 5). The antimicrobial resistance patterns of the isolates from females and the isolates from males were compared, and a marked difference was observed only in doxycycline (p=0.013), with the females having the highest doxycycline-resistant isolates. MRSA (resistance to cefoxitin) was detected in 13 (34.2%) of the 38 isolates, all of which were resistant to three or more agents tested. Isolates with reduced susceptibility to vancomycin (MIC >4 μg/mL) were 14 (36.8%), of which eight were MRSA.

### Multidrug resistance

Multiple drug resistance in this study of healthy volunteers was taken as resistant to three or more antimicrobial drugs, and 25 (65.8%) of the isolates were multidrug-resistant. About one-fourth (23.7%) of the isolates were resistant to four drugs of different resistance combinations. Ampicillin was the most frequently-occurring antibiotic in all the patterns of resistance combinations while co-trimoxazole, doxycycline, and chloramphenicol resistance combinations occurred in only two isolates, and the remaining combinations were all different. The marked difference observed in the multidrug resistance pattern between the isolates from females and those of males in the two groups was not significant (p=0.08) (Table 6).

**Table 1.** Use of antibiotics by study subjects

Type of use	Students (n=60)		Villagers (n=60)		Total (n=120)		p value
	No.	%	No.	%	No.	%	
Use of antibiotics on self-medications	20	33.3	34	56.7	54	45.0	0.01
Use of antibiotics on doctor's prescriptions	40	66.7	26	43.3	66	55.0	0.01

**Fig.**
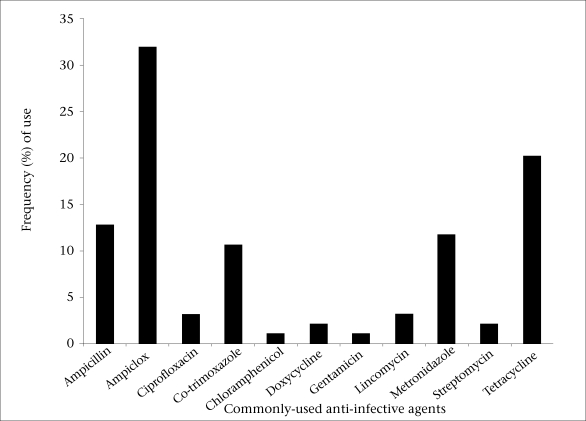
Distribution of frequently-used antimicrobial agents by study subjects

**Table 3.** Patterns of antibiotic use by study subjects

Pattern	Students (n=60)		Villagers (n=60)	
	No.	%	No.	%
Stop drugs when symptoms disappear	17	28.3	24	40.0
Stop when tired of drug	8	13.3	11	18.3
Take some and leave the rest	10	16.7	8	13.3
Take all drugs even when symptoms disappear	25	41.7	10	16.7
Do not complete dose due to financial constraint	0	0.0	7	11.7

The p value for the judicious use of prescribed antibiotics in both the groups=0.003

## DISCUSSION

*S. aureus* remains a versatile and potent pathogen in humans. It is one of the most common causes of nosocomial and community-acquired infections (12). The increasing prevalence of MRSA infections in communities and hospitals is a global health concern, which necessitates measures to reduce or eradicate the colonization of this strain from common sites, such as nares. However, the neglect of the gut, an important reservoir of non-pathogenic antibiotic-resistant *S. aureus* strains, can be a source of re-colonization and a potential source of nosocomial infections (7,13).

**Table 4.** Distribution of intestinal *S. aureus* isolates among study subjects by gender

Sex	No. of samples	No. of *S. aureus* isolates (%)			p value
		Students	Villagers	Total	
Female	60	12	10	22 (36.6)	
Male	60	8	8	16 (26.7)	
Total	120	20 (33.3)	18 (30.0)	38 (31.7)	0.239

**Table 5.** Antimicrobial resistance profile of volunteers' intestinal *S. aureus* isolates

Antimicrobial agent	No. of resistant *S. aureus* isolates						p value
	Overall(n=38)		Students(n=20)		Villagers(n=18)		
	No.	%	No.	%	No.	%	
Ampicillin (10 μg)	26	68.4	16	80.0	10	55.6	0.106
Augmentin (30 μg)	7	18.4	3	15.0	4	22.2	0.566
Cefuroxime (30 μg)	9	23.7	7	35.0	2	11.1	0.084
Gentamicin (10 μg)	2	5.3	1	5.0	1	5.6	0.939
Ciprofloxacin (5 μg)	3	7.9	2	10.0	1	5.5	0.612
Ofloxacin (5 μg)	3	7.9	0	0.0	3	16.7	0.057
Chloramphenicol (30 μg)	13	34.2	4	20.0	9	50.0	0.052
Co-trimoxazole (25 μg)	14	36.8	5	25.0	9	50.0	0.111
Doxycycline* (30 μg)	23	60.5	10	50.0	13	72.2	0.162
Erythromycin (15 μg)	13	34.2	8	40.0	5	27.8	0.428
Cefoxitin (30 μg)	13	34.2	5	25.0	8	44.4	0.207
Vancomycin (30 μg)	14	36.8	8	40.0	6	33.3	0.671

* In the comparison of the differences in bacterial resistance to all the agents between males and females in groups, only the difference in doxycycline resistance was significant (p=0.013)

**Table 6.** Multidrug resistance patterns of *S. aureus* from stools of healthy subjects

Resistance	No. of *S. aureus* isolates (%)						p value
	Females (n=22)		Males (n=18)		Total (n=38)		
	No.	%	No.	%	No.	%	
Fully sensitive	1	4.5	2	12.5	3	7.9	
Resistant to 1 agent	2	9.1	3	18.8	5	13.2	
Resistant to 2 agents	2	9.1	3	18.8	5	13.2	
Resistant to 3 agents	3	13.6	2	12.5	5	13.2	
Resistant to 4 agents	9	40.9	0	0.0	9	23.7	
Resistant to 5 agents	1	4.5	1	6.3	2	5.3	
Resistant to 6 agents	2	9.1	1	6.3	3	7.9	
Resistant to 7 agents	0	0.0	3	18.8	3	7.9	
Resistant to 8 agents	1	4.5	1	6.3	2	5.3	
Resistant to 9 agents	1	4.5	0	0.0	1	2.6	
Resistant to 10 agents	0	0.0	0	0.0	0	0.0	
Resistant to 11 agents	0	0.0	0	0.0	0	0.0	
Resistant to 12 agents	0	0.0	0	0.0	0	0.0	
Resistant to 3 and more agents	17	77.3	8	50.0	25	65.8	0.08

The evaluation of antibiotic use among the study subjects revealed that the villagers practised self-medications of antibiotics and used prescribed antibiotics injudiciously more than the students. The differences were highly significant, which indicate that there are some conditions, such as lack of availability of sufficient health centres with competent personnel, lack of sufficient knowledge on the danger of the wrong use of antibiotics, high proximity to large numbers of unlicensed drug vendors, and high poverty, which predispose them more to the misuse of antibiotic than the students who came from town and cities (14,15). However, the practice of self-medications of antibiotics was generally high (45%) among all the volunteers. This might be as a result of lack of strict antibiotic control in the country, coupled with the high rate of poverty among the people, which usually hinders them from completing the dosage regimen of antibiotics (15,16).

The distribution of the frequently-used anti-infective agents among the subjects showed that ampicillin, ampiclox, co-trimoxazole, metronidazole, and tetracycline were highly misused. This is due to the fact that these agents are readily available to the people at affordable prices in the country. Thus, the excessive use of these agents in an inappropriate dosage regimen will favour the selection of multiple drug-resistant strains that may cause serious infections with limited treatment options (17).

The colonization rate of the gut with *S. aureus* in the study volunteers was observed to be 31.7%. This supports the findings of Bhalla *et al.,* who reported 36.7% of *S. aureus* intestinal colonization in hospitalized patients as against 50.7% nasal *S. aureus* in the same patients (18). There are fewer studies on the prevalence of intestinal *S. aureus* compared to nasal, throat, and skin, which may be as a result of the fact that the gut is not being considered an important reservoir of pathogenic staphylococci. However, several recent studies are now implicating this site as an important source of transmission of antibiotic-resistant *S. aureus-* associated infections to the environmental surfaces of communities and hospitals (19-21). Thus, this calls for more studies on the prevalence of intestinal *S. aureus* in Nigeria and other developing countries where proper sanitary systems may be inadequate in rural areas, as a measure to contain its transmission.

The intestinal isolates of *S. aureus* were highly resistant to ampicillin (68.2%) and doxycycline (60.5%), with the strains from females significantly showing higher resistant to doxycycline than their male counterparts in both the groups (p=0.013). These observations might be due to the extensive use of these agents as observed while evaluating the use of antibiotics by these subjects. The higher resistance to doxycycline observed among females than among males may be due to the excessive and indiscriminate use of the agent in the treatment of pelvic inflammatory disease (22). Hence, the agents may not be useful in the treatment of infections caused by this organism. Chloramphenicol, erythromycin, and co-trimoxazole, which exhibited moderate activities against the strains of this organism, may be useful in combination therapy against infections due to the organism.

Methicillin resistance in the isolates was observed to be 34.2%, which is in contrast to the findings of Currie *et al.* and Baba-Mousa *et al.,* who reported 62% in rectal swabs of hospitalized patients in Canada and 62% in faecal samples of antibiotic-associated diarrhoea patients in Benin respectively (23,24). This marked difference might be due to the varied health status of subjects in the studies. However, the presence of this level of MRSA isolates in stools of these healthy subjects, which exhibited multiple resistances to other agents, is an indication that the gut is an important reservoir of multidrug-resistant *S. aureus*. This might predisposes subjects to antibiotic-associated diarrhoea and other community-associated MRSA infections (25).

The number of isolates of *S. aureus* with reduced susceptibility to vancomycin (VISA) was 14 (36. 8%), with 57.1% of them being MRSA. This is quite higher than findings of Olonitola *et al.* who reported 2.97% in nostrils of healthy children in Zaria, Nigeria (26). Vancomycin resistance was first noticed in *Enterococcus* species which normally colonized the gastrointestinal tract and the co-existence of *S. aureus* with it in the gut might favour the increased emergence of vancomycin-resistant *S. aureus* due to genetic transfer which may account for the observed high prevalence of VISA in this study (27).

The isolates were highly susceptible to cefuroxime, augmentin, ciprofloxacin, ofloxacin, and gentamicin. The observed activities of the stable beta-lactam antibiotics (cefuroxime and augmentin) might be due to their intrinsic characteristics, of being resistant to the beta-lactamases of the organism, coupled with their low level of misuse in the community. *S. aureus* isolates from other sites have been widely reported to be highly susceptible to fluoroquinolones and gentamicin (28,29), which is equally observed in this study. This makes these agents the appropriate drugs of choice in the empirical treatment of most infections caused by this organism since most MRSA and VISA isolates in this study were susceptible to them.

This study reports a high (65.8%) level of multiple antibiotic resistance in intestinal *S. aureus* isolates from the healthy adults, with those from the females higher than the males. Previous studies on other sites of healthy individuals reported a similar high prevalence of multiple antibiotic resistance in *S. aureus* (28,30). This suggests the possibility of having infections due to *S. aureus* with limited treatment options. Hence, there is a need to adopt measures of eradicating and preventing the transmission of this organism to vulnerable populations, especially in women who play a vital role in the transmission.

### Conclusions

The results of this study emphasize the importance of the gut as a reservoir and a source of transmission of multiple antibiotic-resistant strains of *S. aureus* which might lead to more severe infections. Hence, there is a need to adopt strategies to encourage proper personal hygiene and provide adequate effective sewage-disposal systems that will prevent the transmission of these strains to our environments. Also, the need to control the excessive use and misuse of antibiotics as a means of curbing the increasing rate of antibiotic resistance in this country cannot be overemphasized.
